# Modelling of the cancer cell cycle as a tool for rational drug development: A systems pharmacology approach to cyclotherapy

**DOI:** 10.1371/journal.pcbi.1005529

**Published:** 2017-05-03

**Authors:** Robert C. Jackson, Giovanni Y. Di Veroli, Siang-Boon Koh, Ian Goldlust, Frances M. Richards, Duncan I. Jodrell

**Affiliations:** 1 Pharmacometrics Ltd, Cambridge, United Kingdom; 2 Cancer Research UK Cambridge Institute, University of Cambridge, Cambridge, United Kingdom; 3 QCP, Early Clinical Development—Innovative Medicines, AstraZeneca, Cambridge, United Kingdom; Mount Sinai School of Medicine, UNITED STATES

## Abstract

The dynamic of cancer is intimately linked to a dysregulation of the cell cycle and signalling pathways. It has been argued that selectivity of treatments could exploit loss of checkpoint function in cancer cells, a concept termed “cyclotherapy”. Quantitative approaches that describe these dysregulations can provide guidance in the design of novel or existing cancer therapies. We describe and illustrate this strategy via a mathematical model of the cell cycle that includes descriptions of the G1-S checkpoint and the spindle assembly checkpoint (SAC), the EGF signalling pathway and apoptosis. We incorporated sites of action of four drugs (palbociclib, gemcitabine, paclitaxel and actinomycin D) to illustrate potential applications of this approach. We show how drug effects on multiple cell populations can be simulated, facilitating simultaneous prediction of effects on normal and transformed cells. The consequences of aberrant signalling pathways or of altered expression of pro- or anti-apoptotic proteins can thus be compared. We suggest that this approach, particularly if used in conjunction with pharmacokinetic modelling, could be used to predict effects of specific oncogene expression patterns on drug response. The strategy could be used to search for synthetic lethality and optimise combination protocol designs.

## Author summary

Neoplastic transformation results from mutations, chromosomal abnormalities, or expression changes affecting components of the cell cycle, the signalling pathways leading into it, and the apoptosis pathways resulting from cell cycle arrest. Cytotoxic agents, but also newer drugs that target the cell cycle and its signalling pathways, perturb this complex system. Small differences in cell cycle control between normal and transformed cells could determine drug selectivity. Using cell cycle and representative signalling and apoptotic pathway simulations, we examine the influence of cell cycle checkpoints (frequently defective in cancer) on drug selectivity. We show that this approach can be used to derive insights in terms of drug combinations scheduling and selectivity.

## Introduction

Pharmacokinetic and pharmacodynamic (PK/PD) models of anticancer drug action have many potential applications [1–3]. Among the most promising are the ability to match tumours with particular gene expression profiles to selective treatments [4], the ability to search for potential synthetic lethalities [5], and the ability to optimise combination protocols [6]. Thousands of treatment protocols can be screened *in silico*, and the most promising selected for experimental or clinical evaluation [7]. Modelling the cellular pharmacodynamics of anticancer drugs, whether they are cytotoxic agents or targeted agents requires, minimally, a description of three biological processes: the cell cycle, the associated signal transduction pathways, and the apoptotic cascade. There are published models of all of these processes, and our model includes descriptions of the cell cycle, the EGF signalling pathway and apoptosis.

In a previous study we showed that the loss of the G1-S and/or SAC checkpoints were critical to the description of cancer [8]. This was consistent with Duesberg’s theory [9] which suggested that cancer is, in essence, a disease of chromosomal instability. According to this line of thought the phenotypic hallmarks of cancer that arise are the inevitable outcome of the selection process operating on the numerous chromosomal variants.

The evidence linking defective SAC function with cancer has been reviewed by Kops, Weaver and Cleveland [10,11], and by Musacchio and Salmon [12]. There are many deletions or mutations that can cause SAC over-ride, resulting in aneuploidy. One of the commonest SAC abnormalities in human cancer appears to result from over-expression of aurora kinase A [13,14]. Other mitotic proteins whose over-expression or mutation results in aneuploidy include Nek2 [15,16], Hec1 [17], and Mad2 [18]. Our model includes mathematical descriptions of the G1-S and SAC checkpoints where aurora kinase A expression can be manipulated.

There have been a number of attempts to enhance the selectivity of cancer chemotherapy by exploiting loss of checkpoint function in cancer cells, a concept that has been termed “cyclotherapy” [19–21]. Cyclotherapy is an example of new biomarker-driven therapeutic strategies that will require more sophisticated pharmacodynamic modelling to realise their full potential. Here we illustrate how a modelling approach that incorporates the cell cycle oscillator and descriptions of the G1-S and SAC checkpoints, together with EGF signalling and apoptosis pathways, can help in developing such strategies. To study the effects of drugs in various cytokinetic configurations, the sites of action of different anticancer drugs can be incorporated (Table A in S1 Text). We consider here four different drugs: palbociclib, gemcitabine, actinomycin D and paclitaxel. Sets of parameter values can also be used to describe different cell types (Table B in S1 Text). We investigated here with the malignant cell line MiaPaca-2 and normal cell line ARPE-19. Following a brief description of the implementation of this approach, we aim to demonstrate via a series of examples how the strategy can facilitate identifying drug selectivity by simulating drug effects on normal and transformed cells.

## Results

**Fig 1:**
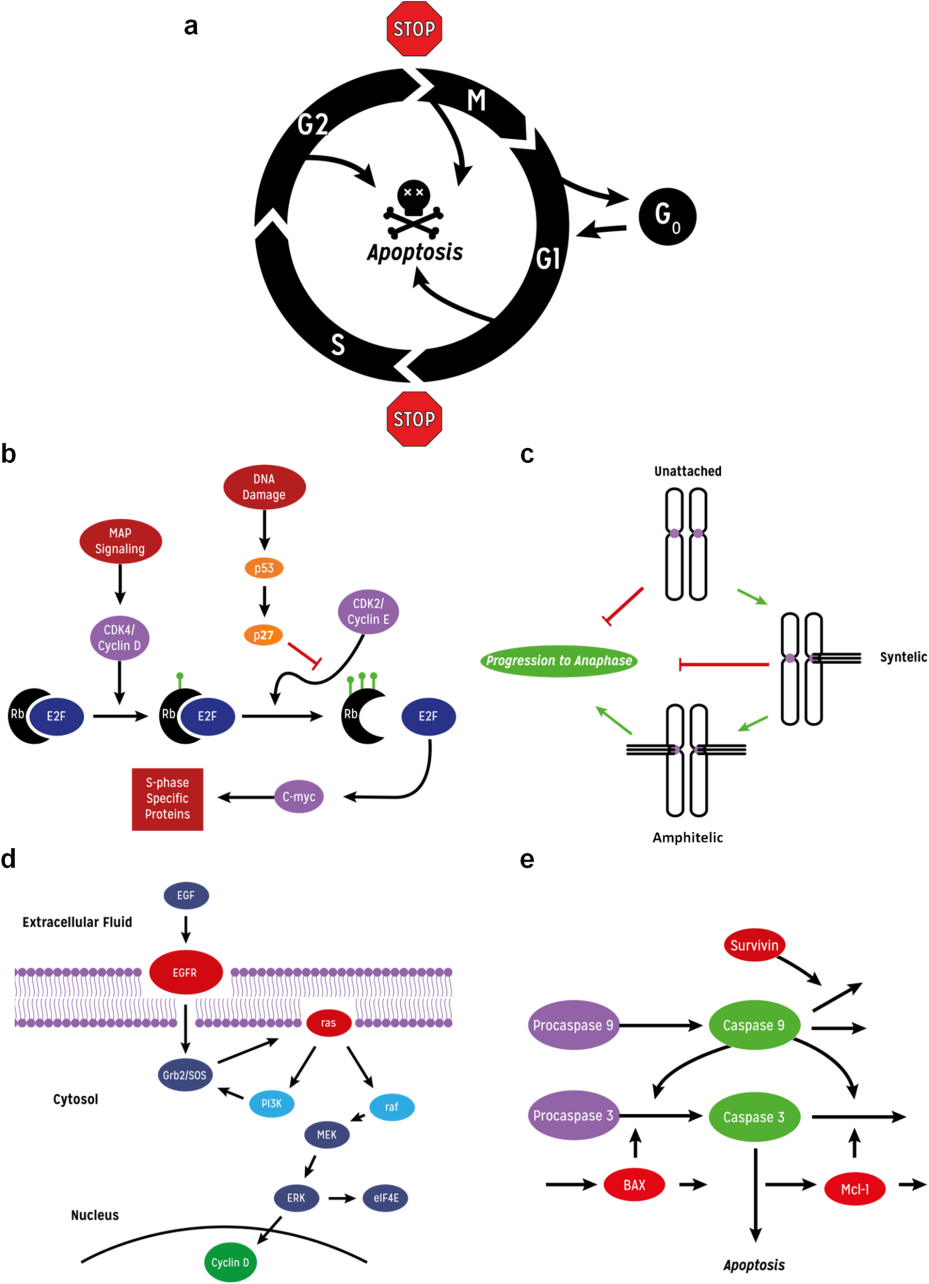
Diagram of global processes and signalling pathways modelled in CYCLOPS. **(a)** Cell cycle partition, checkpoint and apoptosis process. **(b)** Signalling processes modelled in the G1-S and **(c)** spindle assembly checkpoints. **(d)** Signalling modelled in the MAP kinase and **(e)** apoptosis pathways.

**Fig 2:**
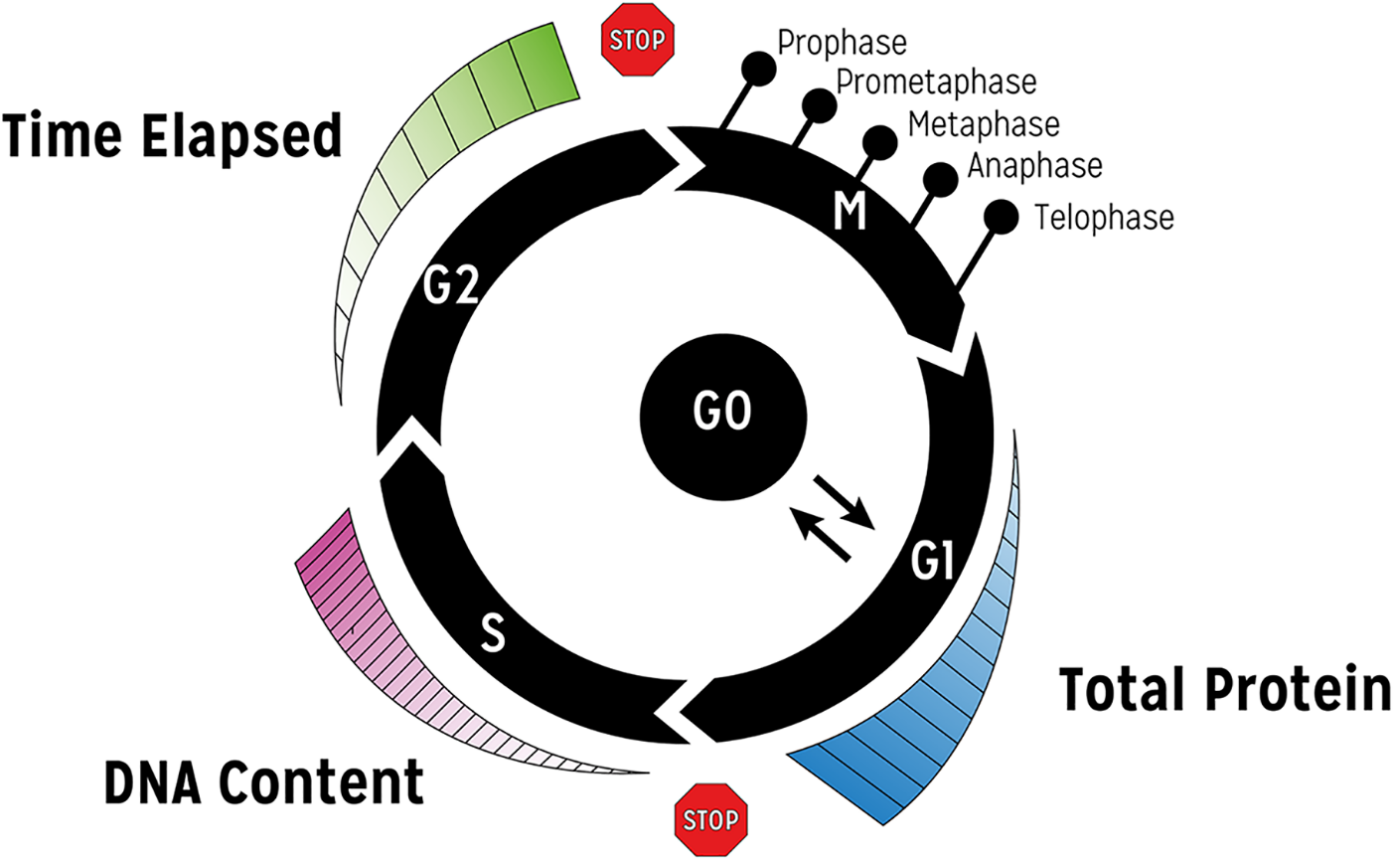
CYCLOPS population approach. Cohorts of cells are modelled at different phases of the cell cycle by taking into account discrete states. Progress through G1 phase is taken into account via increased protein content. Evolution in S phase is considered via increased DNA content. Progress through G2 phase is directly related to time. The M phase is modelled via distinct states. All these phases are modulated via relevant signalling pathways as per Fig 1.

CYCLOPS, is a mathematical model and code which endeavour to investigate CYCLOtherapy Pharmacodynamics Strategies. The basic cell cycle, the G1-S checkpoint, the spindle assembly checkpoint, part of the MAP kinase signal transduction pathway and apoptosis processes are incorporated in this model (Fig 1). Additionally, populations of cells at various stages in the cell cycle are also simulated (Fig 2; see methods for more details). We have used this tool to simulate and compare drug effects on the MiaPaca-2 pancreatic carcinoma cell line and a hypothetical normal cell line that has identical values of the cytokinetic parameters. Normal cells were modelled with basal levels of p53 and AKA, and normal RAS that is only active in the presence of an upstream growth factor. The MiaPaca-2 cells were modelled with constitutively activated mutant RAS and mutant p53 (resulting in override of the G1 checkpoint). Moreover, the MiaPaca-2 cell model included up-regulation of Aurora-kinase-A (AKA) which results in SAC override. We proceed by illustrating basic features of this approach, followed by more elaborated analyses that illustrate how insights in terms of combination strategies can be derived.

### Modulating the cell cycle

**Fig 3:**
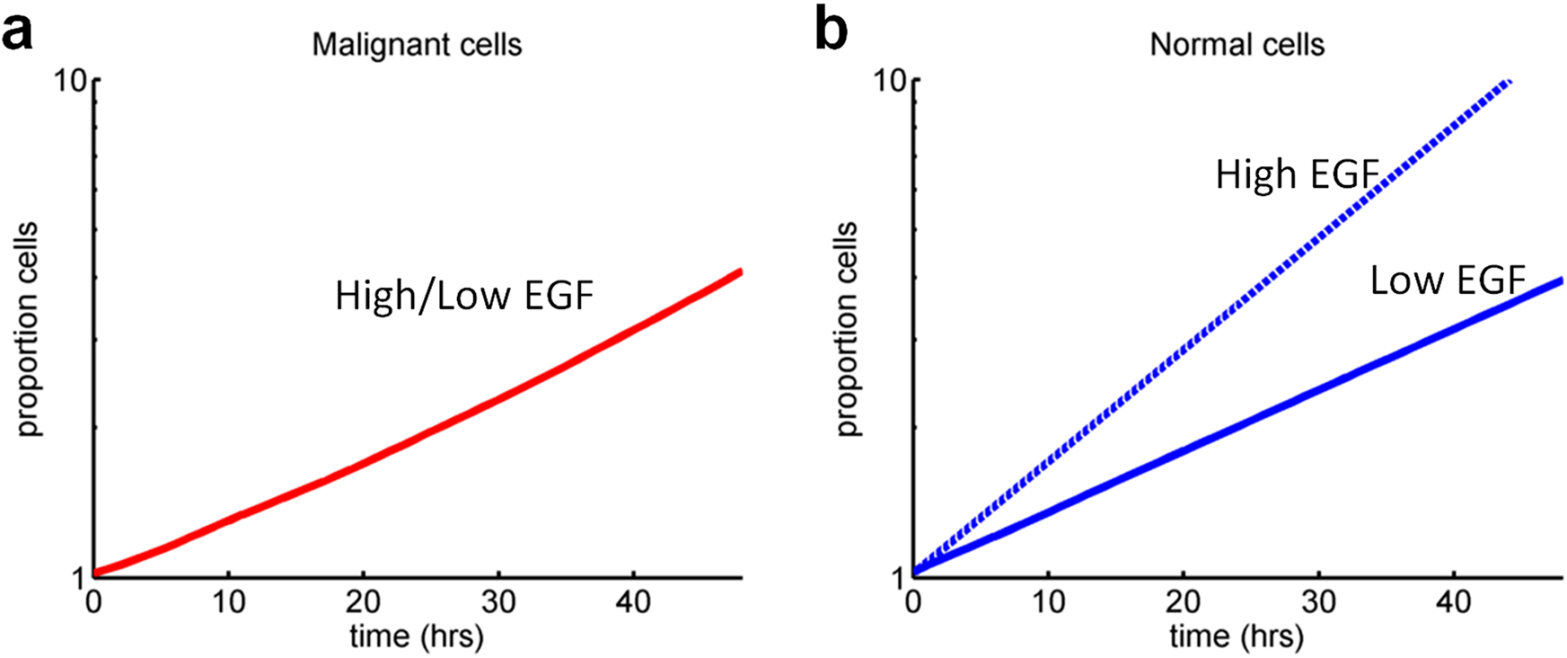
Simulation in CYCLOPS of cell cycle and ligand modulation. EGF stimulation of malignant **(a)** and normal cells **(b)**. “Proportion cells” correspond to the increase in number of cells i.e. 10 = 10 times more cells.

This approach allows the study of cohorts of normal and cancer cells and comparing the effects of drugs. Fig 3 shows the simulated growth of cells in the absence of drug treatment. For normal cells, the cell cycle dynamic can be modulated by the presence of ligands. The model simulations results in normal cells having a shorter doubling time (from 25 to 13 hours; (Fig 3A) while MiaPaca-2 cells, in which MAP kinase signalling downstream from ras was modelled as constitutively activated, were indifferent to EGF concentration (Fig 3B).

### Palbociclib-induced G1 arrest

As mentioned earlier, it has been argued that the selectivity of cancer chemotherapy could be enhanced by exploiting loss of checkpoint function in cancer cells, a concept termed cyclotherapy [19–21]. Since arrest in G1 is non-cytotoxic to normal cells, one approach to cyclotherapy is to treat with a drug that will cause G1 arrest. Cancer cells with impaired G1 checkpoint function, e.g. because of a p53 mutation, will progress into S phase, where they may be selectively killed by an S-phase-specific drug. The cdk4/cdk6 inhibitor palbociclib (PD332991) [22,23] prevents normal cells from progressing through the G1 checkpoint and entering S phase. In cells with mutant ras such as MiaPaca-2 the G1 checkpoint is weakened by high levels of production of cyclin D, making them less prone to arrest following treatment with palbociclib.

**Fig 4:**
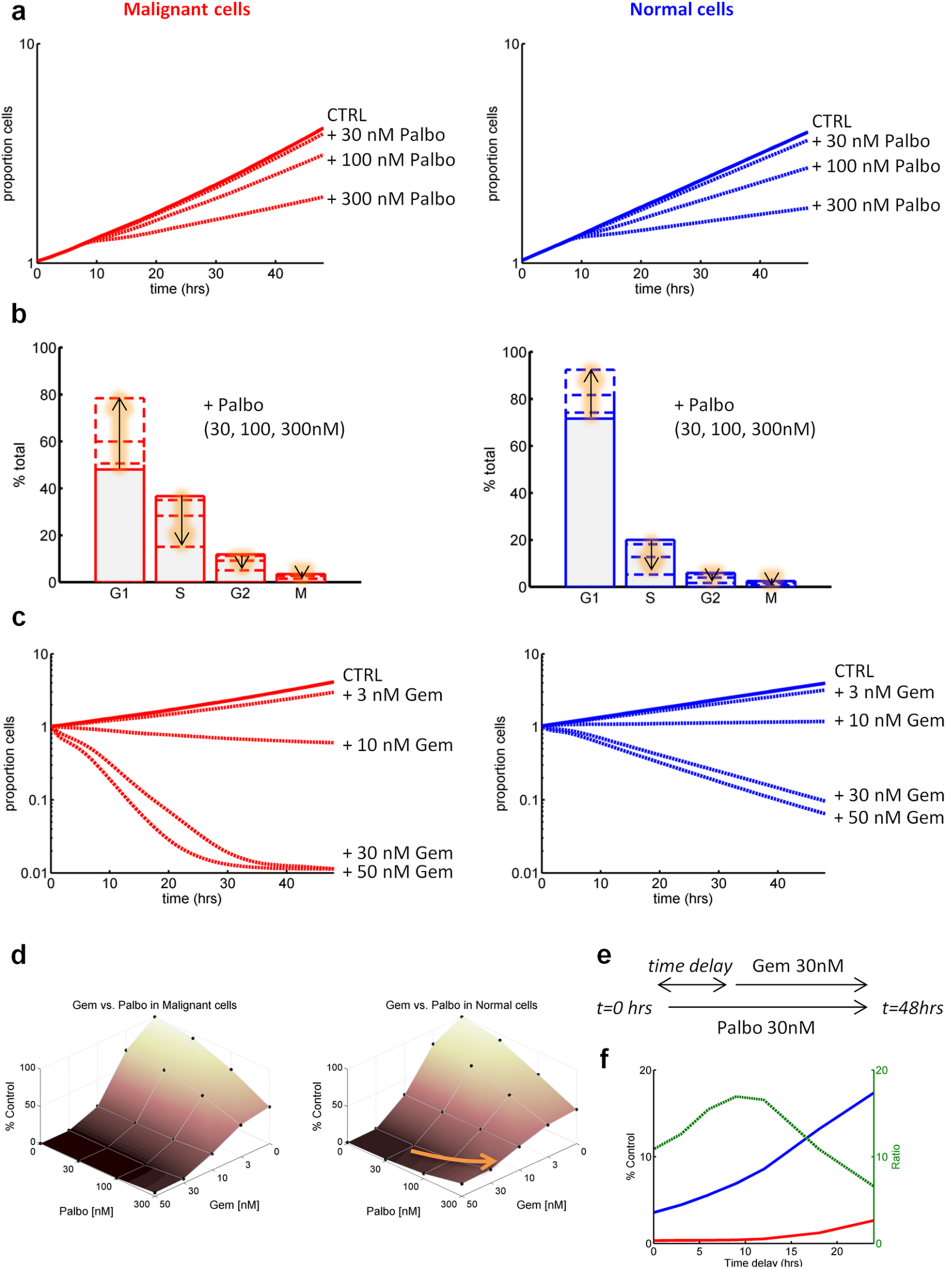
Simulations of palbociclib and gemcitabine effects. **(a)** palbociclib effects on malignant (left) and normal (right) at several concentrations (“proportion cells” correspond to the increase in number of cells i.e. 10 = 10 times more cells). **(b)** Modulation of the cell cycle distribution (left malignant, right normal, arrows indicate changes with increasing concentrations). **(c)** gemcitabine effects on malignant (left) and normal (right) at several concentrations. **(d)** Simulated dose-response surface for the palbociclib+gemcitabine combination. The arrow shows the rise of antagonistic effect with normal cells when increasing palbociclib concentration. **(e)** gemcitabine delayed administration protocol. (f) Effects of varying the time delay on normal (blue line, shown as % control) and malignant cells (red line). The ratio of normal to malignant is shown in green.

Fig 4A shows that the simulated effect of exposure for 48 hours to 30, 100 and 300 nM palbociclib was to reduce proliferation but was non-cytotoxic to both cell lines (thus having minor utility as a single agent at these concentrations). Nevertheless, progression from G1 to S was affected with these concentrations and resulted in a decrease of the population of cells in S phase which was more pronounced in normal cells (Fig 4B).

Combining gemcitabine with palbociclib.

The anticancer drug gemcitabine is selectively cytotoxic to cells in S phase [24]. Gemcitabine might possess inherent anticancer selectivity since cells lacking G1 checkpoint function should have a higher proportion of cells reaching S phase. Fig 4C shows that consistent with this understanding, the model predicted that gemcitabine was more cytotoxic to MIA PaCa-2 cells compared to the normal cell line (Fig 4C).

We then investigated combining gemcitabine with palbociclib since palbociclib was differentially active in allowing cancer cells to enter the S-phase compared to normal cells. The simulated dose-responses showed that the combination affected more malignant cells than normal cells as per gemcitabine alone (Fig 4D). In particular, antagonistic effect was observed with normal cells when adding palbociclib. We then wanted to investigate if delaying gemcitabine administration would enhance differential effect between normal and cancer cell lines. Thus, we simulated cell growth following administration of palbociclib and gemcitabine where gemcitabine administration was delayed (Fig 4E). Concentrations of 30 nM for both drugs were used as these induce similar effects in normal and malignant cells (Fig 4D).

The simulations showed that delaying gemcitabine addition by approximately 12 hours from the start of palbociclib resulted in the best differential effect (Fig 4F), with a ratio of normal cell viability to malignant cells viability of 17. This illustrates from a mechanistic point of view the paradigm of cyclotherapy, in which cells with an intact G1 checkpoint can be selectively protected from cytotoxic agents acting later in the cell cycle. A few experimental studies have demonstrated this effect, generally exploiting the fact that tumour cells with mutant p53 lack a functional G1 checkpoint [25,26]. More advanced optimization approaches can also be employed to attempt the optimisation of both concentration and administration schedules in order to reach one or several pre-defined goals. For instance normal cell viability can be set above a specific threshold or target malignant cell viability can be set below a specific threshold while exposure constraints can also be taken into account.

### Effects of AK-A over expression during paclitaxel induced mitotic arrest

**Fig 5:**
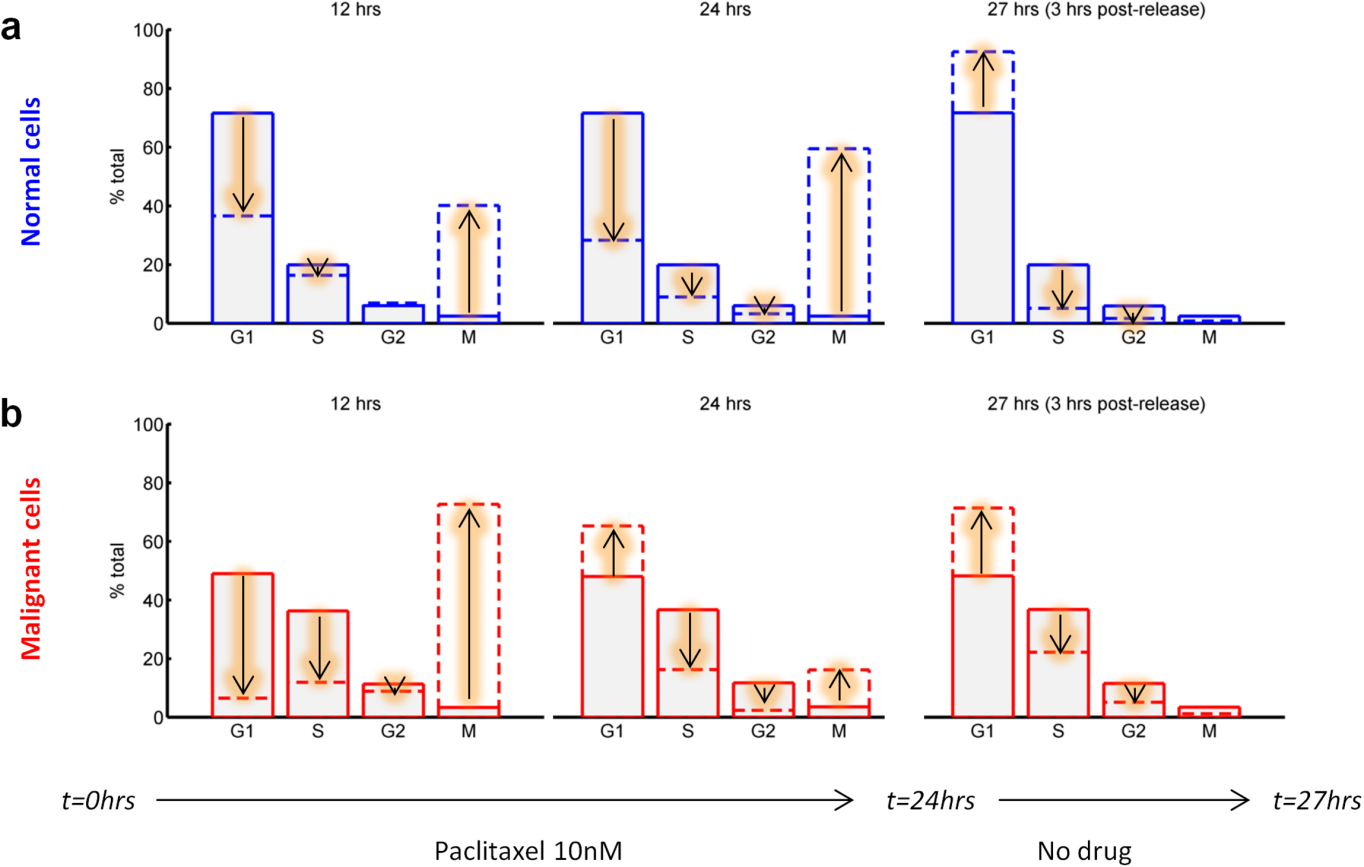
Paclitaxel modulation of the cell cycle distribution. 24hours paclitaxel treatment was simulated and the resulting cell cycle distribution is shown at 12, 24 and 27 hours (3 hours after wash-out) for **(a)** normal and **(b)** malignant cells. Plain bars show the reference cell cycle distribution prior to treatment while broken-line bars show the distribution evolution over time.

In addition to defects in the G1 checkpoint, most tumours may have impaired SAC function. One cause of this is over-expression of AK-A [13,14]. High levels of AK-A tend to be associated with low-level resistance to taxanes [13]. It is not clear, intuitively, whether there is a mechanistic relationship between the AK-A over-expression and the taxane sensitivity. [13] Paclitaxel causes M phase cell cycle arrest, and cells that remain arrested for several hours enter apoptosis. Simulations were used to illustrate how during a 24 hour treatment with 10 nM paclitaxel, cells with a functional SAC accumulate in M phase (Fig 5A). If the drug is removed after 24 hours, a large cohort of cells moved synchronously into G1 (Fig 5A). Malignant cells, which are modelled with higher AK-A levels, also showed an initial increase in the M phase fraction (12 hours; Fig 5B). However, after about 24 hours, the simulation captured the phenomenon of “checkpoint leakiness”, i.e. an increasing number of malignant cells entered cell division even though mitosis was not fully completed, thus appearing in G1 phase (24 hours; Fig 5B). In contrast to normal cells, if the drug was removed after 24 hours, only an additional small cohort of cells move into G1 (Fig 5A).

### Combining actinomycin D and paclitaxel

**Fig 6:**
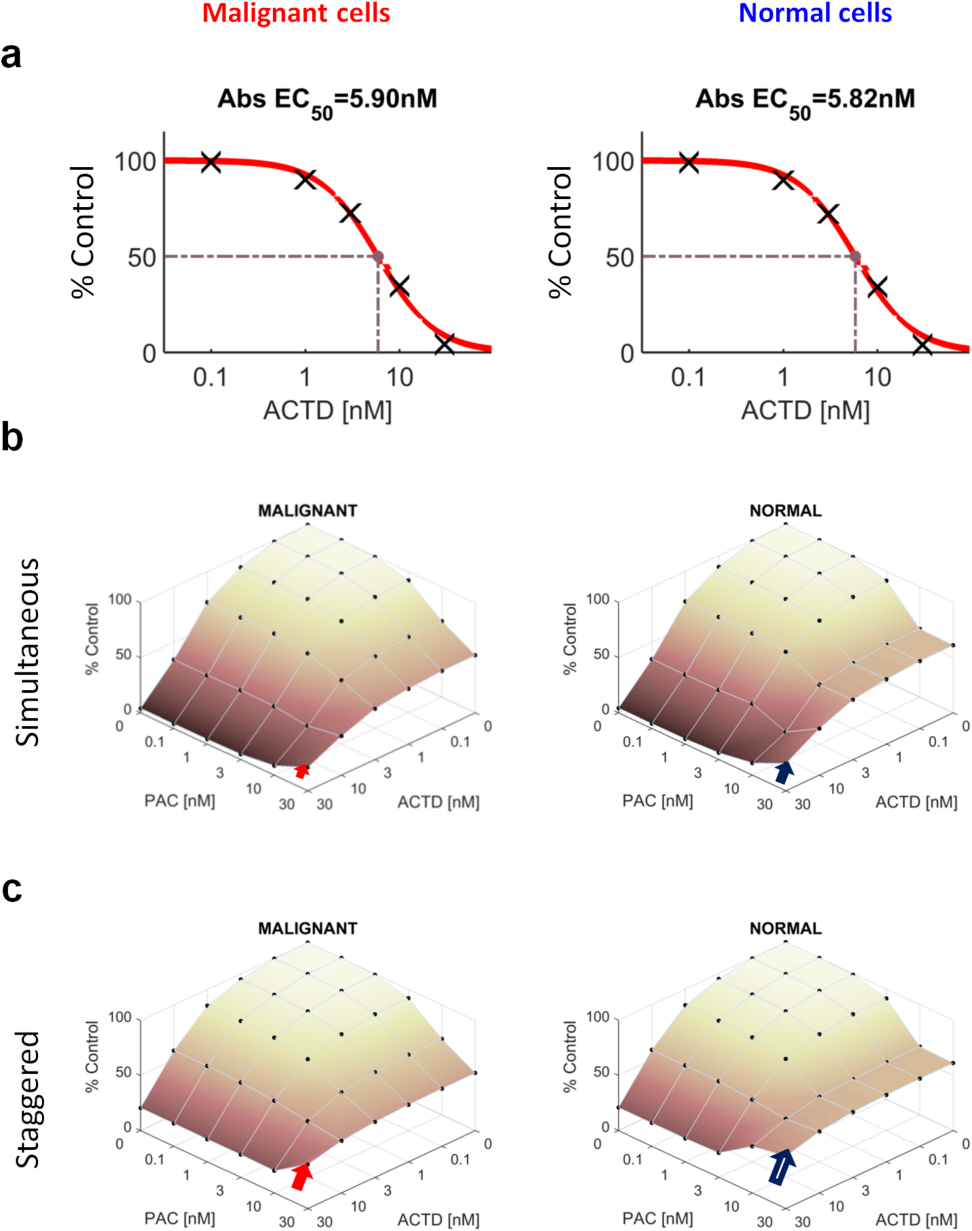
CYCLOPS simulations of actinomycin D and paclitaxel combinations. **(a)** Actinomycin D dose-response are shown for malignant (left) and normal (right) cells. **(b)** Combination dose-response for actinomycin D+paclitaxel for simultaneous administration (24 hours) and **(c)** when delaying actinomycin D by 12 hours (malignant left, normal right). Arrows highlight magnitude of antagonistic effect when adding paclitaxel to 30nM actinomycin D.

Actinomycin D is a transcription inhibitor that kills cells in all phases of the cell cycle except M phase (because transcription is not active during mitosis). Because simulations highlighted a preferential arrest of normal cells in M phase by paclitaxel, we therefore asked the question if combining this drug with actinomycin D could generate preferential kill of tumor versus normal cells. Actinomycin D used as a single agent has similar activity in our modelled malignant and normal cell lines (Fig 6A). We then simulated the combination of actinomycin D with paclitaxel which also resulted in a similar combination dose-response, although with slightly more antagonistic effects for normal cells against malignant ones at the highest concentrations (Fig 6B; antagonistic scores of -17% and -12% respectively). Experiments confirmed differential combination effect, with slight antagonism for normal cells but mild synergy for malignant cells (Fig A in S1 Text).

Earlier, we showed that normal cells tended to accumulate more in M phase in the presence of paclitaxel between 12 and 24 hours (Fig 5), thus suggesting that a combination treatment in which actinomycin D was added after the start of paclitaxel could enhance this differential effect. Further simulations (Fig 6C) showed that antagonistic interactions were enhanced for both cells lines (compared to concomitant treatment, Fig 6B), but indeed with greater protection achieved in normal versus malignant (SAC-deficient) cells (Fig 6B). Nevertheless, the resulting predicted efficacy of the paclitaxel + actinomycin D combination for malignant cells was not strong enough (40% of control) and only marginally better than for normal cells (48% of control) and therefore is not likely to be a therapeutic option. Overall, these results highlight the potential to enhance the therapeutic window by considering inherent dynamical differences between malignant and normal cells.

## Discussion

Several hundred oncogenes and tumour suppressor genes have been identified. What they have in common is that all are involved in control of the cell cycle or its associated signalling pathways (including the apoptosis pathways). [8,25–27]Understanding the dynamics of tumour growth and the pharmacodynamics of anticancer drugs could be greatly assisted by quantitative descriptions of these processes.

It has been argued that malignant transformation involves, minimally, two kinds of somatic mutation. Moreover, it is believed that cancer is a disease of genetic instability[9,10,12,28]and that all human tumours have some degree of aneuploidy. Aneuploidy confers increased spontaneous cell loss, so that tumour cells can only survive and proliferate if they have a compensating growth advantage over competing normal cells. These changes in cancer are usually the result of mutations or changes in expression levels leading to over-ride of cell cycle checkpoints [8,10–12]. Modelling the cell cycle can therefore enable us to capture differences in dynamics between normal and cancer cells resulting from various mutations and associated phenotypes.

Although the essential features of the mammalian cell cycle have been the subject of detailed dynamic models [29–32], the significance of cell-cycle checkpoints has not been well studied. The present model builds upon these available models but also includes detailed kinetic descriptions of two of the major cell cycle checkpoints that are mutated or over-ridden in cancer: the G1 checkpoint and the spindle assembly checkpoint. This approach can be used to explore the potential anti-tumour selectivity of drugs that act on essential components of these checkpoints and the signalling pathways leading to them. This approach might facilitate exploring a therapeutic strategy termed”cyclotherapy”[19,20,33], which attempts to optimise drug selectivity against cells with defective checkpoint function.

The cell cycle, with its associated signalling pathways and apoptotic pathways, constitutes a complex interactive system. Analysing the dynamics of such systems requires that we take into account multiple positive and negative feedbacks, cross-talks, and effects that span multiple spatial compartments and multiple levels of hierarchical organization. Any model that attempts to describe the kinetics of the cell cycle must represent a compromise between this almost intractable complexity and over-simplifications so sweeping that the essential dynamics of the system are lost. The simulations described in this report were chosen to illustrate capabilities and limitations of the suggested approach.

We also provide the tool developed for these studies (CYCLOPS, https://sourceforge.net/projects/cyclops-simulations/). The CYCLOPS model does not incorporate all known biological processes. Only major components of the cell cycle and simplified descriptions of apoptosis, EGF signalling, G1-S and SAC checkpoints are incorporated. Additionally, the model is based on specific kinetics which are only reasonable average values. It should be noted that great cell to cell variability and difficulties in quantifying temporal and spatial profiles of proteins and other cellular components currently excludes deriving models which are truly quantitative.^54^ A more complete description of cell cycle dynamics could also consider the G2-M checkpoint, particularly as there is cross-talk between it and the spindle assembly checkpoint. As illustrated here, models such as CYCLOPS can facilitate the understanding of underlying cellular dynamics and how to develop or optimize therapeutic strategies.

Mechanistic approaches as this one (increasingly termed quantitative systems pharmacology (QSP)[34]) bridges molecular and systems biology studies to traditional PK/PD studies. They offer the potential for accelerating the drug discovery process and making it more cost-effective. An obvious practical application is the design of rational drug combinations for which exhaustive experimental study can be impractical. Potential combinations can be better understood and optimized if assisted by mathematical models. In this context, modelling the cell cycle and its modulating components can facilitate the development of combinations, particularly within the scope of cyclotherapy as illustrated here.

Additional features that can be investigated could include multiple tumour cell populations and a description of mutations from drug sensitivity to resistance, and vice versa. Double mutants with resistance to two drugs can also be modelled. Indeed, another important rationale for combination chemotherapy is the use of combinations of non-cross-resistant drugs to prevent or delay treatment failure resulting from acquired drug resistance. The approach presented here can be extended to facilitate the design and optimization of such combinations.

Although ambitious, it is possible to envision a future where pharmacodynamic models of the cell cycle can also be used to develop personalised chemotherapies. Because each tumour is genetically unique and expressed against a unique genetic background, individualising therapy is essentially a multi-dimensional optimization problem. Eventually, CYCLOPS type and other QSP approaches, together with more traditional PK modelling, might provide powerful tools for matching treatment regimens to each tumour’s particular expression profile.

## Materials and methods

### Modelled processes

We have developed and coded (C language, code available on https://sourceforge.net/projects/cyclops-simulations/) a mathematical model to investigate cyclotherapy pharmacodynamics strategies (CYCLOPS). CYCLOPS allows simulating a cohort of cells in different phases of the cell cycle. For each cell the following processes are modelled: the basic cell cycle, the G1-S checkpoint, the spindle assembly checkpoint, part of the MAP kinase signal transduction pathway and apoptosis. We first describe each one of these cellular processes individually and how they have been incorporated. Then we explain how this model has been used to simulate large cohorts of cells.

### Cell cycle model overview

A comprehensive review of cell cycle modelling has been published by Csikásh-Nagy. [29] Since our underlying premise is that malignant transformation requires, minimally, loss of function of two cell cycle checkpoints, it was necessary to model these checkpoints, and their effects on the pharmacodynamics of anticancer drugs. The CYCLOPS model differs from previous models in incorporating a kinetic description of the spindle assembly checkpoint (SAC) and the G1-S checkpoint (Fig 1A). It is an update of a classical cytokinetic model[35,36] to which has been added a version of the cell cycle oscillator based on that described by Novak and Tyson[30,37] and elaborated by Gérard and Goldbeter [31]. Portions of the cell cycle model have been published in Chassagnole et al.[32]. The description of the mitotic spindle assembly checkpoint is based on that described by Mistry et al. [38] and by Kamei et al.[39].

### The G1- S checkpoint

The normal G1-S checkpoint, as modelled by CYCLOPS, is summarised in Fig 1B. Transcription of most of the enzymes required for DNA replication is driven by the transcription factor c-myc, which in turn is under the control of the transcription factor E2F. E2F in G1 cells is bound to, and inactivated by the RB protein. When the RB protein is phosphorylated, active E2F is released. Phosphorylation of RB is catalysed by the cyclin-dependent kinases, cdk2 and cdk4. Cdk4 is activated by cyclin D, which is produced by a number of signalling pathways, including the MAP kinase pathway. Cdk2 is activated by cyclin E [25].

For progression of cells from G1 phase into S phase it is essential that their DNA is intact and that they have sufficient DNA precursors for DNA synthesis to commence. In the presence of DNA damage, or if the nucleotide pools are depleted or unbalanced, the p53 protein is activated [26]. This results in transcription of p21 and p27, which inhibit cdk2, and thus prevents release of the G1-S checkpoint. When the DNA damage has been repaired, p27 transcription ceases, and the cell enters S phase. If the DNA damage cannot be repaired within a certain time (usually 8 to 24 hours, depending upon cell type) the cell enters apoptosis. About 50% of carcinomas have mutations or deletions of p53 while some tumours lack a functional retinoblastoma (RB) protein, resulting in a dysfunctional G1-S checkpoint. In CYCLOPS, this is modelled via over-ride of the G1-S checkpoint.

Other tumours have mutations that also result in over-ride of the G1-S checkpoint: this can be caused by over-expression of cyclin D or cyclin E, or by mutations that result in constitutive activation of the EGF receptor, or of *ras*. Thus the CYCLOPS model is able to model mechanisms of G1-S checkpoint defect or over-ride. Nevertheless, this model represents a first approximation, and at present does not describe the effects of a number of physiological regulators, such as for instance CDKN2A. The checkpoint model could be elaborated as additional kinetic data becomes available.

### The spindle assembly checkpoint

The SAC acts by sensing correct connections of kinetochores to the two opposite spindle poles. All kinetochores are initially modelled as emitting a “wait” signal. Once all kinetochores reach a state of tension this signal is stopped, thus allowing progression to anaphase (Fig 1C). The wait signal is mediated by the bistable, tension-sensitive Aurora Kinase B (AK-B) [40]. Microtubules grow from the opposite spindle poles, and attach at random to the kinetochores. This results in various configurations which are modelled here:

- Monotelic attachment: the kinetochore of one of a pair of sister chromatids is attached to a single spindle pole.
- Amphitelic attachment: the remaining kinetochore is attached to the opposite spindle pole.
- Syntelic attachment: the remaining kinetochore is attached to the same spindle pole as its sister.

Syntelic attachments are not in a state of tension, which results in the second attachment being removed through activity of the enzyme aurora kinase B. Amphitelic attachments are in a state of tension which results in aurora kinase B being inactive. When all pairs of sister chromatids are correctly attached, the wait signal rapidly decays, and the cell progresses to anaphase. Failure of the SAC results in premature exit from mitosis and aneuploidy. Most tumours are aneuploid, but aneuploidy is never detected in normal replicating cells. [41]

We modelled in CYCLOPS the effects of three drug classes on the SAC. Aurora kinase A (AK-A) inhibition, which slows the process of mitosis by increasing time to anaphase (AK-A is essential for centrosome maturation). Paclitaxel, which stabilises microtubules against depolymerisation and also increases time to anaphase. Aurora kinase B (AK-B) inhibition, which slows down the removal of incorrect microtubule-kinetochore attachments.

### The model of MAP kinase signal transduction

The current model of the MAP kinase pathway used by CYCLOPS is based on the model of Brightman and Fell [42]. Several other groups have modelled this pathway (reviewed in Gilbert et al. [43]). The CYCLOPS model captures much of the essential dynamics of EGF signalling (Fig 1D) and includes sites of action of five classes of drugs. When EGF binds to its cell surface receptor, *RAS* is activated, and signals through RAF, MEK and ERK to up-regulate cyclin D and over-ride the G1-S checkpoint (Fig 1D).

### The model of apoptosis

Caspases are produced as inactive procaspases. One procaspase molecule, when activated (by a cellular damage signal) can then catalytically activate many other procaspase molecules. The process is thus autocatalytic. Like kinases, proteases can act as multi-stage amplifiers. In apoptosis, procaspase 9 is activated to caspase 9, which catalyzes the conversion of procaspase 3 to caspase 3, which is the proximal cause of cell death (Fig 1E). Apoptosis has been modelled mathematically[44–46] and the CYCLOPS model is adapted from these published models.

### Cell populations

To model cancer cytokinetics requires that we can model asynchronous cell populations, which may contain millions of cells. To model the cell cycle oscillator individually in each cell would be impractical. Instead, cells are grouped into a succession of cohorts, assumed to be a few minutes apart. CYCLOPS treats the cell as a sequence of 63 states, with transition rules based upon a combination of elapsed time and biochemical values (Fig 2).

Some of these quantities are modelled continually (DNA, total protein), and others are calculated. In these cohorts, the apparent cell cycle time is modulated by biochemical parameter values. The 63 cytokinetic states are: 15 G1 states (differing in total protein content and cyclin E level), 30 S phase states (differing in DNA content), 10 G2 states (differing in time elapsed from the start of G2), 5 M states (prophase, prometaphase, metaphase, anaphase, telophase), a single G0 phase, a single population of terminally differentiated and senescent cells, and a population of irreversibly damaged cells that are metabolically active but unable to replicate. These 63 compartments can contain any number of cells (Fig 2).

In addition to progressing through the stages of the cell cycle, cells may leave the cycle irreversibly through cell death, differentiation or senescence. Spontaneous cell loss after cell division is treated as a cytokinetic parameter characteristic of particular cell lines, as are rates of differentiation/senescence (Table 1). Senescence, differentiation, and apoptosis may also be stimulated by drug treatment. Cells may leave the cell cycle reversibly and enter a quiescent (G0) compartment (Fig 2).

**Table 1:** Properties of the cell lines modelled. Most values were from the ATCC website.[55] It should be noted that the cytokinetic properties of cell lines vary substantially according to culture medium, concentration of serum and growth factors, inoculum density and oxygen and CO_2_ concentration. The values shown are the ones used in CYCLOPS and may be regarded as typical values for early-stage cultures at low cell density under standard levels of O2 and CO_2_.

Cell line	MiaPaca2 (malignant cell line)	ARPE-19 (“normal” cell line)
Species	human	human
Cell type	pancreatic carcinoma	retinal pigment epithelium
Cell cycle time *	19.5 h	19.8 h
Mean doubling time	~26 h	~26 h
Cell loss factor	0.03	0.05
Quiescent fraction **	0%	15%
Differentiating fraction	0%	20%
P53 status***	mutant	wild-type
Rb status	positive	positive
Ras status	mutant	wild-type
Aurora kinase A	upregulated	baseline

* Cell cycle times can be measured from thymidine labelling experiments, but are often approximated by doubling times at low inocula in rich media.

** Quiescent fractions are calculated from the difference between cell cycle times and measured doubling times.

*** CYCLOPS assumes that p53 is only activated in the presence of DNA damage but the simulations discussed in this first study do not model DNA damage, therefore only RAS and AK-A differences are relevant here.

### Normal vs. cancer cells

In the current study, a modelled MiaPaca-2 cancer cell line was used. A goal of the model is to optimise drug selectivity and the selection of an appropriate normal control cell is essential. Our approach is two-fold: (a) for many anticancer drugs it is possible to identify a particular drug-sensitive normal cell type that represents the site of dose-limiting toxicity. For most anticancer drugs this is bone marrow, intestinal mucosa, skin, or the immune system. There is sufficient cytokinetic information for these tissues to be modelled in detail, and we can describe the effects of many drugs on these tissues explicitly. We then assume that for the purposes of predicting efficacy and selectivity, drug effects on other cell types can be ignored. (b) Our underlying premise is that cancer is primarily a defect of cell cycle checkpoints. For modelling purposes we can then predict anticancer drug selectivity by assuming that normal cells differ from the cancer cell in having fully functional cell cycle checkpoints. Specifically, the MiaPaca-2 cells are modelled as having mutant, constitutively active RAS, resulting in up-regulation of cyclin D, and causing override of the G1 checkpoint^.^[47]. Second, a 3-fold over-production, relative to the normal control of aurora kinase A, causing impaired function of the SAC was also incorporated (Table 1) [13,48,49].

Pharmacodynamic modelling using CYCLOPS.

Four sites of drug action modelled in CYCLOPS were investigated here:

1. Non-cycle-specific cytotoxicity, e.g. actinomycin D [50].
2. S phase-specific cytotoxicity, e.g. gemcitabine [51].
3. Mitotic arrest, e.g. paclitaxel [52].
4. Inhibition of cdk4/cyclin D, resulting in delay of progression from G1 into S phase e.g. palbociclib [22,23].

Drug effects were modelled using a standard Hill equation whose parameter values were obtained from the DrugCARD database [53]. The rate of cytotoxicity was defined as a reduction of the cell count following treatment compared to the count at the start of treatment:

$$Cytotox=100\left(1-\frac{\#cells\;at\;t=24h}{\#cells\;at\;t=0h}\right)$$

### Implementation

CYCLOPS was coded in C and is available online (https://sourceforge.net/projects/cyclops-simulations/). CYCLOPS generates graphical output using the open source program, gnuplot.[54] A flow chart of the model implementation is included in the supplementary material (Fig B in S1 Text). The list of components is given in Supplementary Table B in S1 Text. In the present study the code was compiled using the free gcc compiler, release 4.6.2.

## Supporting information

S1 Text Includes Supplementary Materials and Methods, Supplementary Figs A and B, Supplementary Tables A and B and Supplementary References.
(DOC)
