# Supplementary material for: Modelling of the cancer cell cycle as a tool for rational drug development: A systems pharmacology approach to cyclotherapy
Source: PLoS Comput Biol. 2017 May 3;13(5):e1005529. doi: 10.1371/journal.pcbi.1005529 (PMC5435348; doi:10.1371/journal.pcbi.1005529)
Supplement: S1 Text — Includes Supplementary Materials and Methods, Supplementary Figs A and B, Supplementary Tables A and B and Supplementary References. (DOC) [file pcbi.1005529.s001.doc]

**SUPPLEMENTARY MATERIAL for:**

**Modelling of the cancer cell cycle as a tool for rational drug development: a systems pharmacology approach to cyclotherapy**

**Supplementary Materials and Methods**

*Cell culture*

MiaPaca-2 cells were obtained from the American Type Culture Collection (ATCC). Cells were grown in DMEM medium (Life Technologies) supplemented with 5% fetal calf serum at 37ºC and 5% CO_2_. The immortalized human retinal pigment epithelial cell line ARPE-19 was obtained from the European Collection of Cell Cultures (ECACC; Health Protection Agency, Salisbury, UK) and was grown in DMEM:F12 medium supplemented with 10% fetal calf serum at 37ºC and 5% CO_2_. Both lines were verified by STR genotyping and tested negative for mycoplasma. Actinomycin D and paclitaxel were obtained from Tocris Bioscience (Bristol, UK). They were dissolved in dimethylsulphoxide (DMSO) and then diluted in culture medium to a final concentration of 0.2% DMSO.

*Cytotoxicity assay*

Drug cytotoxicity in vitro was assessed by the means of sulforhodamine B colorimetric (SRB) assay. Cells were plated in a 96 well plate and treated with a range of concentrations of actinomycin D from 0.1 nM to 50 nM in columns and paclitaxel from 0.03 nM to 30 nM in rows, giving a grid of 8x8 concentration combinations. After 72h of incubation at 37°C, cells were fixed (3% trichloroacetic acid in water (w/v), 90 minutes, 4°C), washed in water and stained with a 0.057% SRB (Sigma) solution in acetic acid (w/v) for 30 minutes. The plates were washed (1% acetic acid (v/v), 4 times), and the protein-bound dye was dissolved in a 10 mM Tris base solution (pH 10.5). Fluorescence was measured using a Tecan Infinite M200 plate-reader (excitation 488 nm, emission 585 nm). Percentage inhibition compared to solvent control-treated cells was calculated for each drug concentration combination and the results saved in a .xls file for each biological replicate (N=4). The data were analysed using Combenefit.^1^

**Supplementary Figure A:** Experimental combination dose-response for actinomycin D and paclitaxel (please see supplementary materials and methods for more details).


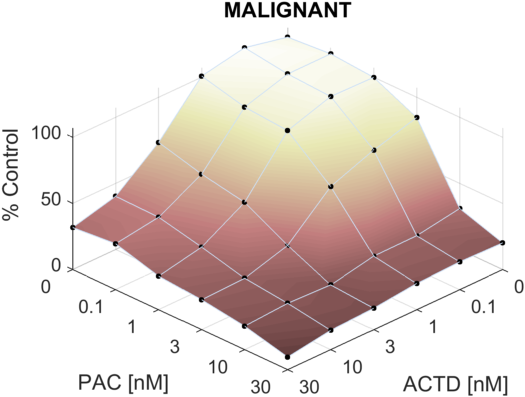

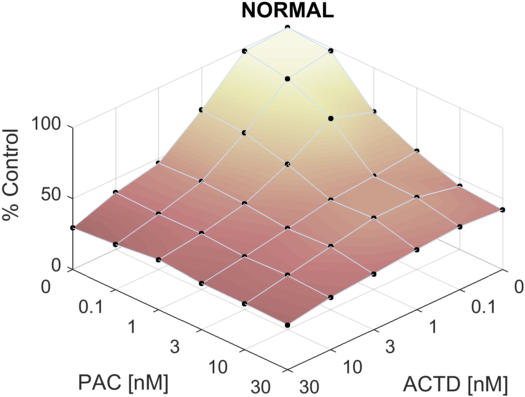


**Supplementary Figure B:** Flow chart of the CYCLOPS model. Notes to the chart:

1. PK model: output is table of drug concentrations (up to 3 drugs) with time, which can be accessed by the cell cycle model. For in vitro modelling, drug concentrations can be constant for defined time periods.

2. MAP kinase pathway: output is level of cyclin D (in normal and transformed cells)

3. G1 checkpoint status: function of cyclin D, Rb status, p53 status and cdk4 activity

4. Movement of cells between cell cycle compartments, as function of cytokinetic parameters, time, checkpoint function, and drug concentrations.

5. SAC activity: possible outcomes are mitotic arrest, normal exit, or premature arrest (resulting in aneuploidy)

6. Population transitions (mutations assumed to occur during mitosis): drug sensitivity to resistance (or vice versa); primary to metastatic. Metastasis: function of primary tumour size, metastatic potential, antimetastatic drugs.

7. Calculation of spontaneous cell loss (greater in aneuploid cells)

8. Angiogenesis: Function of tumour size, distance from nearest capillary. Effect of antiangiogenic drugs.

9. Transitions in and out of cycle. Proportion of quiescent cells, as function of tumour size, hypoxia, and oxygen availability

10. Apoptosis : time-dependent caspase activation. May occur from any phase of the cell cycle

11. T cell-mediated cytotoxicity: immune stimulants

12. Calculate tumour size (primary and secondary). Sum size of normal cell populations (bone marrow, GI mucosa). Evaluate whether endpoints reached: cure, lethal tumour size, lethal toxicity.


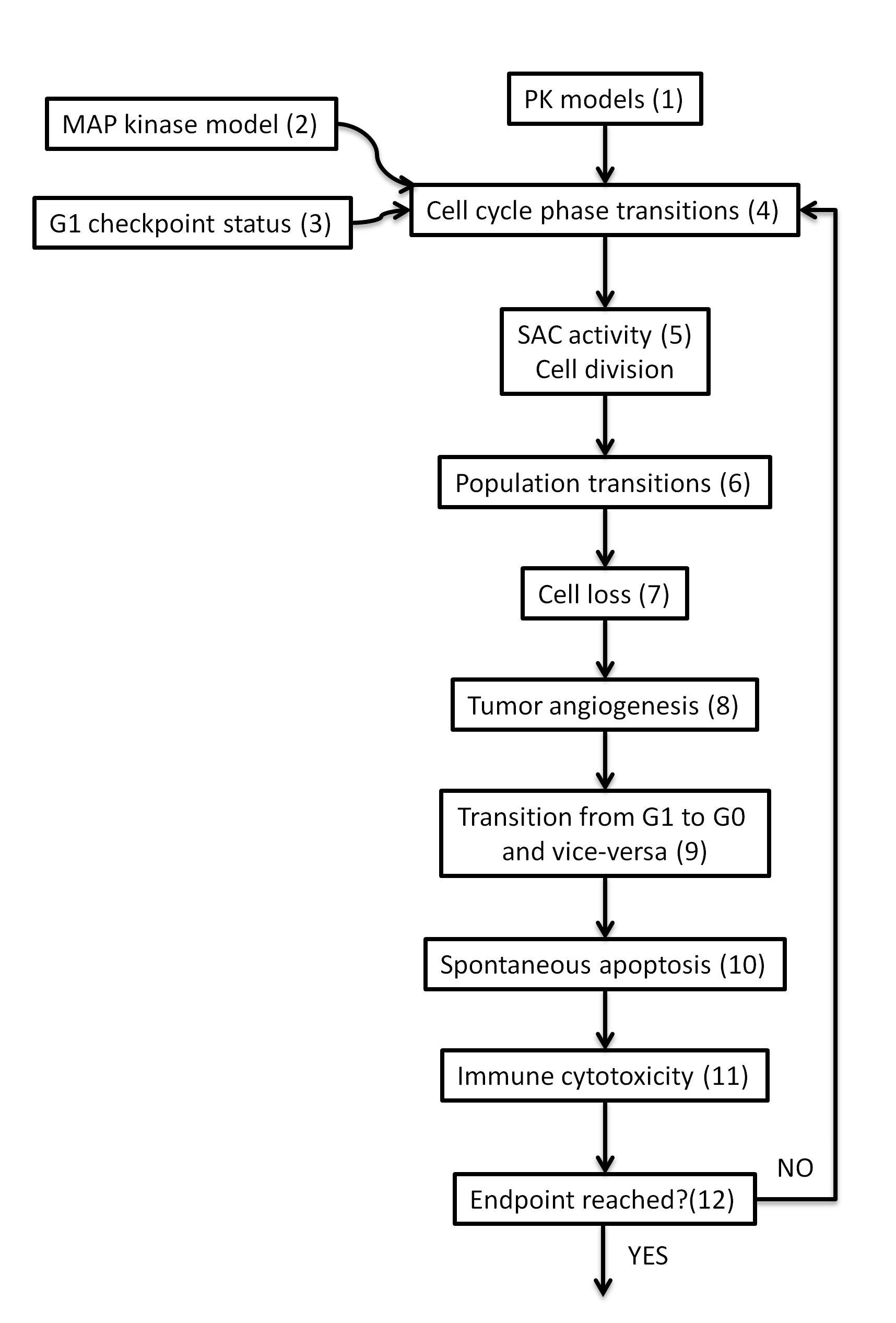


**Supplementary Table A: Drug Targets Included in the CYCLOPS Model.** The underlying idea of the approach taken with the CYCLOPS model is that the cell cycle, its checkpoints, the signalling pathways leading into it, and the apoptosis pathways leading out of it, form a single complex interactive system. To understand cell cycle dynamics it is necessary to include, at least in outline, a description of all these components, and of drugs that act on them. Our cyclotherapy example is an in vitro study and most of the drugs listed here are not discussed in this initial study. Nevertheless CYCLOPS can also model (in conjunction with PK models) the pharmacodynamics of in vivo treatment (parts of the system, in particular angiogenesis and immunomodulation, are only relevant in the in vivo context). These alternative approaches to cancer treatment will be considered in future studies (references are provided only for drugs which are not well known, but that have been added due to the availability of kinetics data).

__________________________________________________________________________

Target System Molecular site of action Drugs

___________________________________________________________________________

Angiogenesis Cortexolone^55^

Cell cycle G1 phase protein synthesis 8-azaguanine

cdk2 BMI1026

cdk4 palbociclib

Cell cycle G2 phase topoisomerase 2 doxorubicin

Cell cycle S phase DNA chain termination 2'-cyano-2'deoxy-arabinofuranosylcytosine, cytarabine, gemcitabine

Dihydrofolate reductase methotrexate, trimetrexate

Cell differentiation induction hexamethylene-bis-acetamide

DNA replication cross-linking carmustine (BCNU), ceemustine (CCNU)

cisplatin, melphalan

Nonspecific Immune levamisole

Stimulator

MAP kinase signalling MEK AZD8330

Ras GTPase FPTIII^49^

EGFR-TK erlotinib

Metastasis indomethacin^56^

Mitosis aurora kinase A MLN8237

Aurora kinase B CYC116

Tubulin depolymerisation docetaxel, paclitaxel

Tubulin polymerisation vincristine

PDGF signalling PDGFR, KIT, ABL TKs imatinib

PI3 kinase signalling PI3 kinase LY294002

Akt perifosine

mTOR rapamycin

RNA transcription cdk9 flavopiridol, seliciclib, actinomycin-D

___________________________________________________________________________

**Supplementary Table B: List of components modelled in CYCLOPS**

Diploid DNA content 6.1 mg/10^9^ cells

Total protein 600 mg/10^9^ cells

Aurora kinase A 3.0 IU/10^9^ cells

Aurora kinase B 1.0 IU/10^9^ cells

BAX 33.7 nM

Caspase 3 0 IU/10^9^ cells

procaspase 3 5.2 IU/10^9^ cells

Caspase 9 0 IU/10^9^ cells

procaspase 9 12.5 IU/10^9^ cells

CDK1 9.3 IU/10^9^ cells

CDK2 0.01 IU/10^9^ cells

c-myc 0.39 nM

cyclin B 10.0 nM

cyclin D 0.339 nM

cyclin E 271 nM

E2F 1.0 nM

EGF 1.2 nM

EGFR-TK 0.33 IU/10^9^ cells

Grb2 0.00595 IU/10^9^ cells

K-ras 5.49 IU/10^9^ cells

Mcl-1 0.0 nM

MEK 40.0 IU/10^9^ cells

phospho-MEK 2.12 IU/10^9^ cells

Microtubules (unattached) 46.0 nM

microtubules (correctly attached) 0.0 nM

Microtubules (syntelically attached) 0.0 nM

microtubules (merotelically attached) 0.0 nM

p16 0.0 nM

p21 0.0 nM

p27 0.0 nM

p53 1.0 nM

RB 1.0 nM

**Supplementary References**

1. Di Veroli, G. Y. *et al.* Combenefit: an interactive platform for the analysis and visualization of drug combinations. *Bioinforma.*  (2016). doi:10.1093/bioinformatics/btw230
